# Supplementary material for: Parental Prepuberty Overweight and Offspring Lung Function
Source: Nutrients. 2022 Apr 4;14(7):1506. doi: 10.3390/nu14071506 (PMC9002985; doi:10.3390/nu14071506)
Supplement: Supplementary file 1 [file nutrients-14-01506-s001.zip › nutrients-1624826-supplementary.pdf]

ONLINE SUPPLEMENTARY MATERIAL

Title: Parental prepuberty overweight and offspring lung function

Marianne Lønnebotn[*,†,1,2], Lucia Calciano[†,3], Ane Johannessen[1], Deborah L. Jarvis[4,5], Michael J. Abramson[6], Bryndís Benediktsdóttir[7], Lennart Bråbäck[8], Karl A. Franklin[9], Raul Godoy[10], Mathias Holm[11], Christer Janson[12], Nils O. Jõgi[13], Jorunn Kirkeleit[1,2], Andrei Malinovschi[14], Antonio Pereira-Vega[15], Vivi Schlünssen[16,17], Shyamali C. Dharmage[‡,18], Simone Accordini[‡,3], Francisco Gómez Real[‡,13,19], Cecilie Svanes[‡,1,2]

[*]Corresponding author. [†]Equal contribution as first authors. [‡]Equal contribution as last authors.

[1]Centre for International Health, Department of Global Public Health and Primary Care, University of Bergen, Bergen, Norway; [2]Department of Occupational Medicine, Haukeland University Hospital, Bergen, Norway; [3]Unit of Epidemiology and Medical Statistics, Department of Diagnostics and Public Health, University of Verona, Verona, Italy; [4]Faculty of Medicine, National Heart & Lung Institute, Imperial College, London, United Kingdom; [5]MRC-PHE Centre for Environment and Health, Imperial College, London, United Kingdom; [6]School of Public Health & Preventive Medicine, Monash University, Melbourne, Australia; [7]Faculty of Medicine, University of Iceland, Reykjavik, Iceland; [8]Section of Sustainable Health, Department of Public Health and Clinical Medicine, Umeå University, Umeå, Sweden; [9]the Department of Surgical and Perioperative Sciences, Surgery, Umeå University, Umeå, Sweden; [10]Department of Pulmonary Medicine, University Hospital Complex of Albacete, University of Castilla La Mancha, Albacete Spain; [11]Occupational and Environmental Medicine, School of Public Health and Community Medicine, Institute of Medicine, Sahlgrenska Academy, University of Gothenburg, Sweden; [12]Department of Medical Sciences: Respiratory, Allergy and Sleep Research, Uppsala University, Uppsala, Sweden;

[13]Department of Clinical Science, University of Bergen, Bergen, Norway; [14]Department of Medical Sciences: Clinical Physiology, Uppsala University, Uppsala, Sweden; [15] Pneumology Service. Juan Ramón Jiménez University Hospital in Huelva, Spain; [16] Department of Public Health, Environment, Work and Health, Danish Ramazzini Centre, Aarhus University, Denmark; [17]National Research Center for the Working Environment, Copenhagen, Denmark; [18]Allergy and Lung Health Unit, School of Population and Global Health, University of Melbourne, Melbourne, Australia; [19]Department of Obstetrics and Gynaecology, Haukeland University Hospital, Bergen, Norway.

CORRESPONDING AUTHOR:

Marianne Lønnebotn, MSc, PhD, Centre for International Health, Department of Global Public Health and Primary Care, University of Bergen, Postbox 7804, N-5020 Bergen, Norway; phone: +47 95968484; e-mail: marianne.lonnebotn@uib.no.

Table S1. Distribution by RHINESSA study centre in the paternal and maternal lines.

		Paternal line		Maternal line	
**Country**	**Centre**	**N° of offspring**	**N° of fathers**	**N° of offspring**	**N° of mothers**
Denmark	Aarhus	18	17	2	2
Estonia	Tartu	24	21	66	51
Iceland	Reykjavik	41	33	61	50
Norway	Bergen	182	119	168	118
Sweden	Goteborg	20	19	32	30
	Umea	34	27	44	33
	Uppsala	39	32	45	39
Spain	Albacete	9	5	10	8
	Huelva	12	7	32	19
Australia	Melbourne	41	28	50	38
	**Total**	**420**	**308**	**510**	**388**

Table S2. Natural direct effects* on offspring's pre-bronchodilator FEV_1/FVC (model 2) within the paternal line.

	Offspring's overweight before puberty‡ beta (95%CI)		Offspring's adult height (cm) beta (95%CI)		Offspring's FEV_1/FVC beta (95%CI)	
	Sons	Daughters	Sons	Daughters	Sons	Daughters
Fathers' overweight (*vs* never)						
before puberty	0.55 (-0.19, 1.19)	0.83 (0.32, 1.45)	**-3.41 (-6.18, -0.57)**	-2.11 (-4.59, 0.64)	0.97 (-1.9, 4.14)	-0.84 (-2.94, 1.19)
at age 30 years but not before puberty	0.07 (-4.26, 0.88)	-0.02 (-0.79, 0.57)	-0.28 (-3.56, 6.85)	-0.2 (-2.73, 1.86)	0.24 (-3.97, 3.57)	-0.02 (-2.71, 2.44)
Offspring's overweight before puberty	-	-	0.38 (-1.17, 1.99)	**-1.16 (-2.28, -0.09)**	-0.13 (-1.4, 1.05)	-0.2 (-1.17, 0.79)
Offspring's height in adulthood (cm)	-	-	-	-	-0.12 (-0.23, 0.01)	-0.09 (-0.21, 0.02)

* Difference (beta) in offspring's expected lung function value for the change in exposure status, keeping offspring's height and/or offspring's overweight at their expected value when the exposure is absent. Model 2 also includes the potential confounders and adjusting variables of the mediators (fathers' low education level) and of the outcomes (fathers' low education level and offspring's age and ever smoking). Beta is a probit regression coefficient for the effect on the latent mediator (offspring's overweight before puberty) and a linear regression coefficient for the effect on offspring's adult height and FEV1/FVC.

‡ Offspring's overweight before puberty was considered as a continuous latent mediator variable.

95%CI: 95% confidence interval. The statistically significant effects are indicated in bold.

Table S3. Natural indirect effects* on offspring's pre-bronchodilator FEV_1/ FVC (model 2) within the paternal line.

	Indirect effects	Offspring's FEV_1/FVC beta (95%CI)	
		Sons	Daughters
Father's overweight (*vs* never)			
before puberty	via offspring's overweight	-0.07 (-1.11, 0.68)	-0.17 (-1.18, 0.73)
	via offspring's height	0.4 (-0.05, 0.95)	0.2 (-0.08, 0.68)
	via offspring's overweight and height	-0.02 (-0.21, 0.08)	0.09 (-0.03, 0.33)
at age 30 years but not before puberty	via offspring's overweight	-0.01 (-1.54, 3.16)	0 (-0.37, 0.45)
	via offspring's height	0.03 (-0.77, 0.52)	0.02 (-0.19, 0.35)
	via offspring's overweight and height	0 (-0.13, 0.64)	0 (-0.12, 0.1)

* Difference (beta) in offspring's expected lung function value when the exposure is present, but offspring's height and/or offspring's overweight change from their expected value when the exposure is absent to their expected value when the exposure is present. Model 2 also includes the potential confounders and adjusting variables of the mediators (fathers' low education level) and of the outcomes (fathers' low education level and offspring's age and ever smoking).
95%CI: 95% confidence interval. The statistically significant effects are indicated in bold.

Table S4. Natural direct effects* on offspring's pre-bronchodilator FEV_1/FVC (model 2) within the maternal line.

	Offspring's overweight before puberty[‡] beta (95%CI)		Offspring's adult height (cm) beta (95%CI)		Offspring's FEV_1/FVC beta (95%CI)	
	Sons	Daughters	Sons	Daughters	Sons	Daughters
Mothers' overweight (*vs* never)						
before puberty	0.16 (-0.42, 0.67)	0.23 (-0.15, 0.58)	-1.74 (-3.91, 0.44)	-1.73 (-3.55, 0.20)	0.39 (-1.33, 2.09)	1.58 (0.02, 3.15)
at age 30 years but not before puberty	0.13 (-0.45, 0.62)	-0.05 (-0.53, 0.37)	-0.43 (-2.70, 1.79)	-0.67 (-2.74, 1.44)	0.01 (-2.01, 2.02)	0.87 (-0.86, 2.59)
Offspring's overweight before puberty	-	-	0.33 (-0.99, 1.59)	-0.37 (-1.49, 0.73)	-0.12 (-1.16, 0.97)	0.22 (-0.64, 1.12)
Offspring's height in adulthood (cm)	-	-	-	-	**-0.14 (-0.25, -0.02)**	-0.01 (-0.12, 0.10)

* Difference (beta) in offspring's expected lung function value for the change in exposure status, keeping offspring's height and/or offspring's overweight at their expected value when the exposure is absent. Model 2 also includes the potential confounders and adjusting variables of the mediators (mothers' low education level) and of the outcomes (mothers' low education level and offspring's age and ever smoking). Beta is a probit regression coefficient for the effect on the latent mediator (offspring's overweight before puberty) and a linear regression coefficient for the effect on offspring's adult height and FEV_1/FVC.

‡ Offspring's overweight before puberty was considered as a continuous latent mediator variable.

95%CI: 95% confidence interval. The statistically significant effects are indicated in bold.

Table S5. Natural indirect effects* on offspring's pre-bronchodilator FEV_1/ FVC (model 2) within the maternal line.

	Indirect effects	Offspring's FEV_1/FVC beta (95%CI)	
		Sons	Daughters
Mother's overweight (*vs* never)			
before puberty	via offspring's overweight	-0.02 (-0.39, 0.37)	0.05 (-0.21, 0.38)
	via offspring's height	0.24 (-0.06, 0.70)	0.02 (-0.19, 0.26)
	via offspring's overweight and height	-0.01 (-0.08, 0.06)	0.00 (-0.02, 0.03)
at age 30 years but not before puberty	via offspring's overweight	-0.02 (-0.39, 0.31)	-0.01 (-0.31, 0.21)
	via offspring's height	0.06 (-0.28, 0.42)	0.01 (-0.13, 0.15)
	via offspring's overweight and height	-0.01 (-0.09, 0.04)	0.00 (-0.02, 0.02)

* Difference (beta) in offspring's expected lung function value when the exposure is present, but offspring's height and/or offspring's overweight change from their expected value when the exposure is absent to their expected value when the exposure is present. Model 2 also includes the potential confounders and adjusting variables of the mediators (mothers' low education level) and of the outcomes (mothers' low education level and offspring's age and ever smoking).
95%CI: 95% confidence interval. The statistically significant effects are indicated in bold.

Table S6. Natural indirect effects* on offspring's post-bronchodilator FEV_1 and FVC (model 1) or FEV_1/ FVC (model 2) within the paternal line.

	Indirect effects	Offspring's FEV_1 (mL) beta (95%CI)		Offspring's FVC (mL) beta (95%CI)		Offspring's FEV_1/FVC beta (95%CI)	
		Sons	Daughters	Sons	Daughters	Sons	Daughters
Father's overweight (*vs* never)							
before puberty	via offspring's overweight	1 (-77, 86)	17 (-40, 86)	8 (-81, 114)	28 (-37, 111)	-0.04 (-1.19, 0.86)	-0.21 (-1.14, 0.65)
	via offspring's height	**-165 (-312, -29)**	-71 (-163, 21)	**-223 (-410, -41)**	-96 (-221, 27)	0.34 (-0.11, 0.96)	0.23 (-0.07, 0.77)
	via offspring's overweight and height	10 (-37, 89)	-23 (-77, 6)	14 (-49, 120)	-32 (-102, 9)	-0.02 (-0.23, 0.09)	0.08 (-0.03, 0.31)
at age 30 years but not before puberty	via offspring's overweight	0 (-233, 118)	-4 (-48, 29)	1 (-339, 102)	-7 (-58, 33)	-0.01 (-1.87, 2.80)	0.05 (-0.43, 0.62)
	via offspring's height	0 (-153, 317)	15 (-86, 90)	0 (-210, 429)	20 (-118, 117)	0.00 (-0.7, 0.45)	-0.05 (-0.33, 0.36)
	via offspring's overweight and height	1 (-262, 48)	6 (-14, 51)	2 (-358, 65)	8 (-19, 68)	0.00 (-0.11, 0.54)	-0.02 (-0.19, 0.05)

* Difference (beta) in offspring's expected lung function value when the exposure is present, but offspring's height and/or offspring's overweight change from their expected value when the exposure is absent to their expected value when the exposure is present. Model 1 and model 2 also include the potential confounders and adjusting variables of the mediators (fathers' low education level) and of the outcomes (fathers' low education level and offspring's age and ever smoking).
95%CI: 95% confidence interval. The statistically significant effects are indicated in bold.

Table S7. Natural indirect effects* on offspring's post-bronchodilator FEV_1 and FVC (model 1) or FEV_1/ FVC (model 2) within the maternal line.

	Indirect effects	Offspring's FEV_1 (mL) beta (95%CI)		Offspring's FVC (mL) beta (95%CI)		Offspring's FEV_1/FVC beta (95%CI)	
		Sons	Daughters	Sons	Daughters	Sons	Daughters
Mother's overweight (*vs* never)							
before puberty	via offspring's overweight	7 (-35, 55)	7 (-13, 39)	8 (-42, 67)	9 (-15, 48)	-0.01 (-0.41, 0.40)	-0.02 (-0.31, 0.22)
	via offspring's height	-77 (-179, 19)	-67 (-138, 3)	-109 (-256, 26)	-86 (-176, 4)	0.23 (-0.05, 0.70)	0.12 (-0.06, 0.41)
	via offspring's overweight and height	3 (-18, 30)	-4 (-23, 7)	5 (-25, 43)	-5 (-29, 9)	-0.01 (-0.10, 0.06)	0.01 (-0.02, 0.05)
at age 30 years but not before puberty	via offspring's overweight	6 (-38, 51)	-3 (-34, 20)	7 (-44, 60)	-4 (-45, 23)	-0.01 (-0.40, 0.37)	0.01 (-0.20, 0.31)
	via offspring's height	-22 (-129, 79)	-18 (-102, 64)	-31 (-182, 115)	-23 (-128, 81)	0.06 (-0.27, 0.43)	0.03 (-0.14, 0.24)
	via offspring's overweight and height	3 (-14, 33)	2 (-13, 18)	4 (-20, 47)	2 (-17, 23)	-0.01 (-0.10, 0.05)	0.00 (-0.04, 0.03)

* Difference (beta) in offspring's expected lung function value when the exposure is present, but offspring's height and/or offspring's overweight change from their expected value when the exposure is absent to their expected value when the exposure is present. Model 1 and model 2 also include the potential confounders and adjusting variables of the mediators (mothers' low education level) and of the outcomes (mothers' low education level and offspring's age and ever smoking).
95%CI: 95% confidence interval. The statistically significant effects are indicated in bold.

Figure S1. Graphical representation of the mediation model for FEV_1/FVC in sons or daughters within the paternal or maternal lines. (*Model 2*).

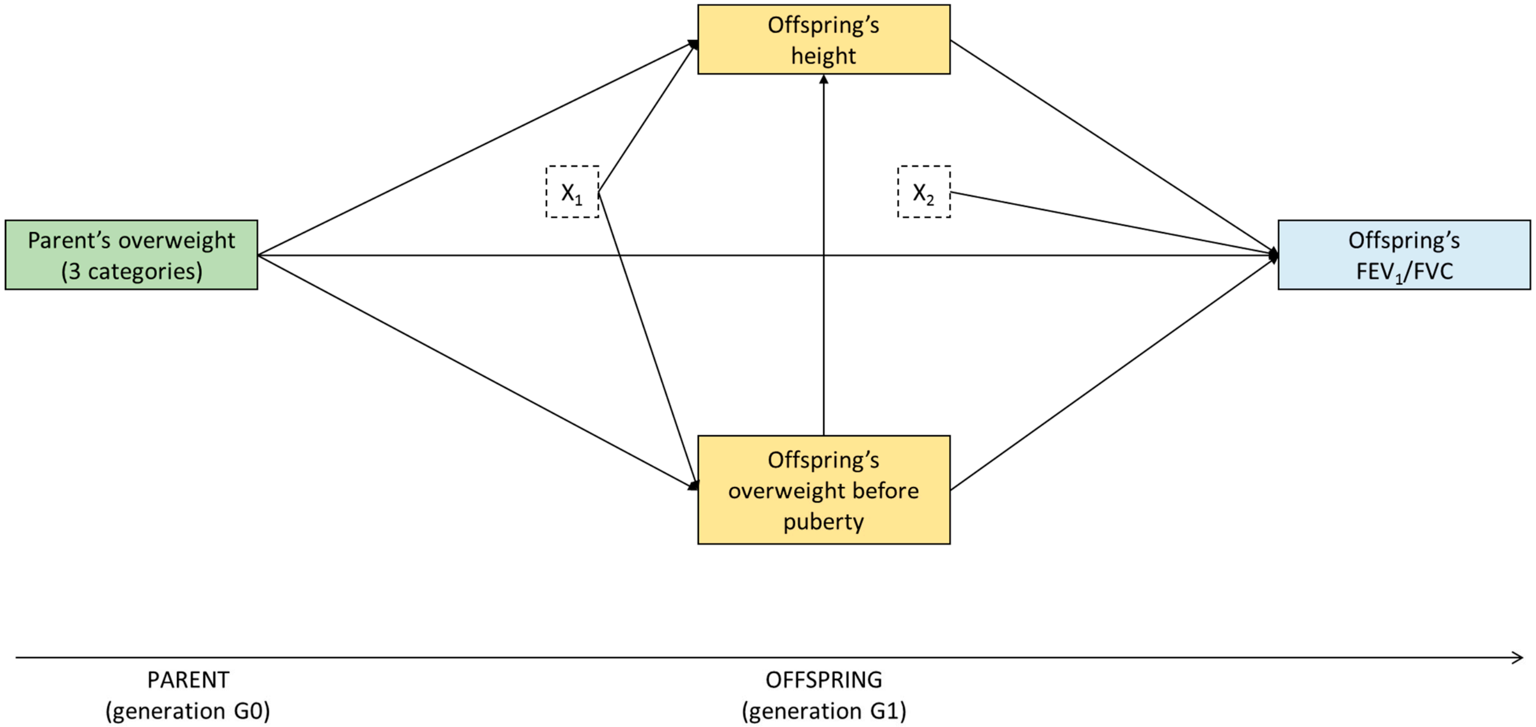

The green box represents the exposure of interest, the yellow boxes the mediators and the blue boxes the outcomes. The dotted boxes represent the set of potential confounders and adjusting variables of the mediators (X_1: parent's low education level) and of the outcomes (X_2: parent's low education level and offspring's age and smoking).

Figure S2. Proportion of Monte Carlo simulations where results match (solid line) and average absolute difference (dashed line) in the average direct (black line) and indirect (red line) effects of fathers' overweight start in prepuberty on sons' lung function (whether one or two unmeasured confounders are included or excluded from the models). Outcomes: (a) FEV_1 and (b) FVC.

One unmeasured confounder | **Two unmeasured confounders**

(a)

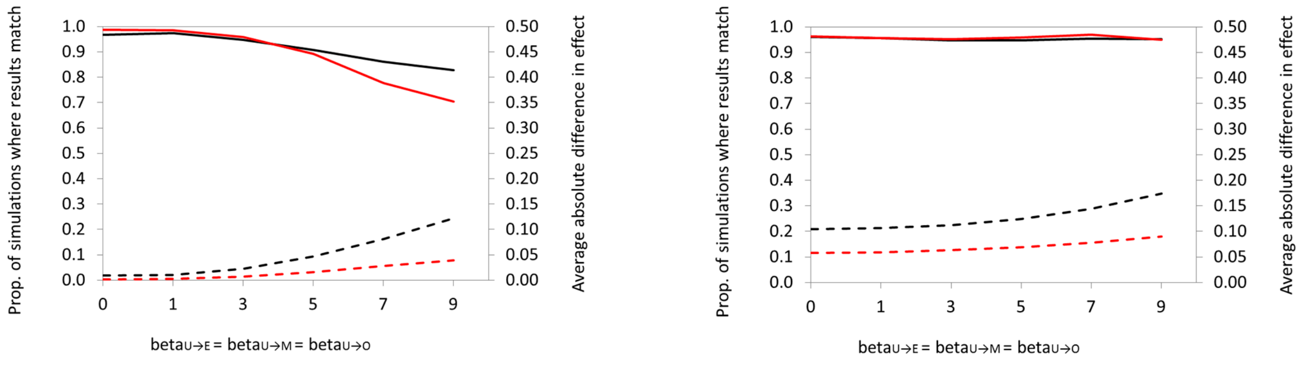

(b)

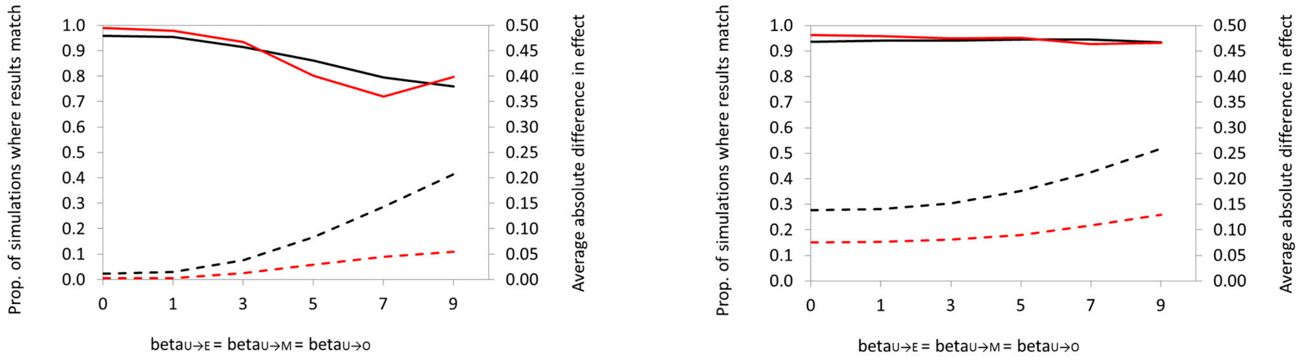

SUPPLEMENTARY INFORMATION ON THE EUROPEAN COMMUNITY RESPIRATORY HEALTH SURVEY (ECRHS)

ECRHS III

Principal Investigators and senior scientific teams: Australia: Melbourne (M. Abramson, G. Benke, S. Dharmage, B. Thompson, S. Kaushik, M. Matheson). **Estonia: Tartu** (R. Jõgi, H.Orru). **Iceland: Reykjavik** (T. Gislason, B. Benediktsdottir, D. Gislason, E.S. Arnardottir, M. Clausen, G. Gudmundsson, L. Gudmundsdottir, H. Palsdottir, K. Olafsdottir, S. Sigmundsdottir, K. Bara-Jörundsdottir). **Norway: Bergen** (C. Svanes, E. Omenaas, A. Johannessen, T. Skorge, F. Gomez Real). **Spain: Albacete** (J. Martinez-Moratalla Rovira, E. Almar, A. Mateos, S. García, A. Núñez, P. López, R. Sánchez, E. Mancebo), **Huelva** (J. Antonio Maldonado, A. Pereira, J. Luis Sánchez, L. Palacios), Oviedo (F. Payo, I. Huerta, N. Sánchez, M. Fernández, B. Robles). **Sweden: Göteborg** (K. Torén, M. Holm, J-L. Kim, A-C. Olin, A. Dahlman-Höglund), **Umea** (B. Forsberg, L. Braback, L. Modig, B. Järvholm, H. Bertilsson, K.A. Franklin, C. Wahlgreen), **Uppsala** (B. Andersson, D. Norback, U. Spetz Nystrom, G. Wieslander, 10 G.M. Bodinaa Lund, K. Nisser). UK: London (P. Burney, D. Jarvis, S. Kapur, R. Newson, J. Potts), Ipswich (N. Innes), Norwich (A. Wilson). **Denmark: Århus** (Torben Sigsgaard-team, Vivi Schlünssen, Christine Cramer, Anne Mette Würtz).

Financial Support: Australia: Melbourne: National Health & Medical Research Council; **Estonia: Tartu:** Estonian Ministry of Education (SF0180060s09); **Iceland: Reykjavik:** The Landspitali University Hospital Research Fund, University of Iceland Research Fund, ResMed Foundation, California, USA, Orkuveita Reykjavikur (Geothermal plant), Vegagerðin (The Icelandic Road Administration (ICERA). **Norway: Bergen:** Norwegian Research council (214123), Western Norway Regional Health Authorities (911631), Bergen Medical Research Foundation. **Spain:** Fondo de Investigación Sanitaria (PS09/02457, PS09/00716, 09/01511, PS09/02185, PS09/03190), Servicio Andaluz de Salud, Sociedad Española de Neumología y Cirurgía Torácica (SEPAR 1001/2010), Fondo de Investigación Sanitaria (PS09/02457); **Huelva:** Fondo de Investigación Sanitaria (FIS PS09/02185), Servicio Andaluz de Salud; **Sweden:** (All centres) The Swedish Heart and Lung Foundation, The Swedish Asthma and Allergy Association, The Swedish Association against Lung and Heart Disease, Swedish Research Council for health, working life and welfare (FORTE); **Göteborg:** Swedish Council for Working life and Social Research; **Umea:** Vasterbotten Country Council ALF grant.

Coordination: The coordination was funded through the Medical Research Council (92091).

Ethics approval: Ethics approval was obtained by all centres from the appropriate ethics committees: **Tartu:** Research Ethics Committee of the University of Tartu, Estland; **Reykjavik:** National Biotecs Committe of Iceland (NBCI) (N° VSNb2011090016/03.11); **Bergen:** Universitetet i Bergen, Regional komité for medisinsk og helsefaglig forskningsetikk, Vest-Norge (REK Vest) (N° 2010/759); **Albacete:** Comité de Ética e Investigación de Complejo Hospitalario de Albacete (N° 04/09); **Huelva**: Comisión de Investigación del Hospital Juan Ramón Jiménez de Huelva (N° 20090417); **Swedish centres:** Ethics Committee at the Medical Faculty, Uppsala University (N° 1999/313 and 2010/068); **Århus:** De videnskabsetiske komiteer for region Midtjylland (M-20110106).
